# Evaluation of Nebulised Dexmedetomidine Given Pre-operatively to Attenuate Hemodynamic Response to Laryngoscopy and Endotracheal Intubation: A Randomised Control Trial

**DOI:** 10.7759/cureus.25223

**Published:** 2022-05-22

**Authors:** Priyanka Shrivastava, Mukesh Kumar, Saket Verma, Ridhima Sharma, Raman Kumar, Ravi Ranjan, Jay Prakash

**Affiliations:** 1 Anaesthesiology, Rajendra Institute of Medical Sciences, Ranchi, IND; 2 Biochemistry, Trauma Centre, Rajendra Institute of Medical Sciences, Ranchi, IND; 3 Anaesthesiology, Post Graduate Institute of Child Health, Noida, IND; 4 Critical Care Medicine, Rajendra Institute of Medical Sciences, Ranchi, IND

**Keywords:** laryngoscopy, intubation, hemodynamic response, nebulised, dexmedetomidine

## Abstract

Background and aim

A variety of medications have been studied to reduce the hemodynamic response to laryngoscopy and intubation. Dexmedetomidine has been used intravenously in many studies to reduce the hemodynamic response to laryngoscopy and intubation. In high-risk patients, this pressor response can increase morbidity and mortality. As dexmedetomidine has a good bioavailability via the nebulisation route, we formulated this study to evaluate the effect of nebulised dexmedetomidine on the hemodynamic response to laryngoscopy and endotracheal intubation.

Methods

This is a prospective, randomised controlled trial conducted on 100 patients with the American Society of Anesthesiologists grade I and II. The primary objective of the study was to see if nebulised dexmedetomidine at a dose of 1 microgram/kg could reduce the stress reaction to laryngoscopy and intubation. The secondary objective was to study the dose sparing effect of nebulised dexmedetomidine on the amount of propofol used during induction of general anaesthesia. The study population was randomly divided into two groups: group A (n = 50) included patients nebulised with dexmedetomidine 1 microgram/kg and group B (n = 50) included patients nebulised with 5 ml saline 30 minutes before induction of anaesthesia in a sitting position.

Results

The demographics were similar in both groups. Following laryngoscopy and intubation, the systolic blood pressure (SBP), diastolic blood pressure (DBP), mean arterial pressure (MAP), and heart rate showed a significant increase in the control group B as compared to the treatment group A. In group A, there was attenuation in SBP (one minute = 113.2 ± 14.503, P < 0.001; five minutes = 109.86 ± 8.342, P < 0.001; 10 minutes = 114.24 ± 7.797, P = 0.010), DBP (one minute = 73.72 ± 10.986, P = 0.011; five minutes = 71.62 ± 9.934, P = 0.005; 10 minutes = 76.1 ± 8.006, P = 0.009), MAP (one minute = 86.80 ± 11.86, P = 0.001; five minutes = 84.44 ± 8.97, P = 0.006; 10 minutes = 88.72 ± 7.44, P = 0.018), and heart rate (one minute = 83.34 ± 12.325, P = 0.001; five minutes = 81.56 ± 13.33, P = 0.003; 10 minutes = 80.16 ± 14.086, P = 0.013) following laryngoscopy and intubation. Induction dose of propofol was significantly lower in the dexmedetomidine group (73 ± 19.509, P < 0.001).

Conclusion

Nebulised dexmedetomidine effectively blunts the hemodynamic response to laryngoscopy and intubation and also has a dose sparing effect on the induction dose of propofol.

## Introduction

Direct laryngoscopy and intubation are associated with a hemodynamic response, which is characterised by an increase in blood pressure and heart rate. This response occurs within 30 seconds after intubation and lasts for less than 10 minutes [1]. These transient responses are normally harmless in healthy people, but they can be dangerous in patients with reactive airways, hypertension, coronary artery disease, myocardial insufficiency, and cerebrovascular disorders [2]. Numerous drugs such as opioids, beta-blockers, and intravenous lignocaine are used to attenuate this hemodynamic response.

Dexmedetomidine is an alpha-2 agonist with sedative, sympatholytic, amnestic, and analgesic actions [3]. Dexmedetomidine in various doses and routes, such as intravenous [4], intranasal [5,6], intramuscular [7], and nebulised [8,9], had been studied to reduce hemodynamic response to intubation. In paediatric patients, nebulised dexmedetomidine in doses of 1-2 microgram/kg has been found to be effective for premedication [10].

The nasal mucosa accounts for 65% of the bioavailability of nebulised dexmedetomidine, while the buccal mucosa accounts for 82% [11]. Intravenous dexmedetomidine has been associated with hypotension and bradycardia, so an alternative route of nebulisation was thought to be explored.

The primary outcome was to evaluate the role of nebulised dexmedetomidine 1 microgram/kg as a premedication in attenuating the stress response to laryngoscopy and intubation. The secondary objective was to study the dose sparing effect of nebulised dexmedetomidine on the amount of propofol used during induction of general anaesthesia.

## Materials and methods

This study was conducted after obtaining approval from the Institutional Ethics Committee, Rajendra Institute of Medical Sciences, Ranchi (letter number: 323). This trial was registered on the Clinical Trials Registry, India (CTRI/2021/08/035888).

Inclusion criteria

This study is a double-blinded randomised controlled trial. After taking informed written consent, 100 patients with the American Society of Anesthesiologists grade I and II and a normal airway undergoing elective surgery under general anaesthesia with endotracheal intubation were included in the study.

Exclusion criteria

Patients with anticipated difficult intubation and those requiring more than 15 seconds for intubation or more than one attempt at laryngoscopy, patients having an allergy to dexmedetomidine, taking medicines that affect the heart rate such as clonidine and beta-blockers, pregnant patients, and patients undergoing emergency surgeries were excluded from the study.

Method

A day before surgery, all patients were visited. They were fasted for the night before surgery and given tablet ranitidine 150 mg and tablet alprazolam 0.5 mg orally as pre-medication.

Patients were randomly divided into two groups (A and B) by computer-generated random numbers. The drug for nebulisation was prepared by an anaesthesiologist not involved in the study. Allocation concealment was done by using sequentially numbered opaque sealed envelopes, which were opened in the operating room by the anaesthesiologist who prepared the drug. Patients were taken to the operating table and all essential monitors were attached like blood pressure, heart rate, and pulse oximetry. All these parameters were continuously monitored during the nebulisation procedure. Group A received nebulisation with dexmedetomidine in the dose of 1 microgram/kg made to a total volume of 5 ml by mixing normal saline. Group B was nebulised with 5 ml 0.9% normal saline. An electric compressor nebuliser, which generated fine mist and turned the full volume to mist in 15-20 minutes, was used for nebulisation 30 minutes prior to induction of general anaesthesia in a propped up position at 45 degrees. Incidence of bradycardia, hypotension, or sedation was noted. Sedation was assessed by the Observer's Assessment of Alertness/Sedation Scale [12]. Fall in systolic blood pressure (SBP) > 20% of the baseline was treated with an injection of mephentermine 3 mg bolus, and in case of bradycardia, i.e., heart rate < 50/minute, it was treated with IV atropine 0.6 mg bolus dose. In case of hypotension (SBP < 20% of baseline) or bradycardia (heart rate < 50/minute), nebulisation was stopped.

Application of bi-spectral index sensors was done before preoxygenation. All patients were preoxygenated for three minutes. After 1 mg midazolam and 2 microgram/kg fentanyl premedication, 1-2 mg/kg propofol was administered in 10 mg aliquots titrated to the loss of vocal responsiveness. After achieving adequate bag and mask ventilation, vecuronium 0.10 mg/kg was given, and ventilation was done with a bag and mask for three minutes. Depth of anaesthesia was achieved with isoflurane in 50% oxygen in the air to obtain a bi-spectral index of 50-60. End-tidal carbon dioxide monitoring was done and the value was maintained at 32-35 mmHg.

Anaesthesiologists with at least 10 years of experience in anaesthesia performed the intubation. Any intubation taking more than 15 seconds was excluded from the study. Direct laryngoscopy was performed and endotracheal intubation was done using an appropriate size endotracheal tube. Then controlled ventilation was started by connecting the patient to the ventilator. The patient was left undisturbed for 10 minutes and parameters such as SBP, diastolic blood pressure (DBP), mean arterial pressure (MAP), and pulse oximetry were recorded at the baseline, after nebulisation, and one, five, and 10 minutes after intubation by an anaesthesia resident who was not involved in the study. After the operation, all patients were given 1 gm paracetamol and 50 mg tramadol intravenously. At the end of the surgery, residual neuromuscular blockade was reversed with a 10 microgram/kg injection of glycopyrrolate and 0.05 microgram/kg neostigmine intravenously, and the trachea was extubated on meeting the extubation criteria.

Statistical testing was conducted using SPSS version 21.0 (IBM Corp., Armonk, NY). Continuous variables are presented as mean ± SD, and categorical variables are presented as absolute numbers and percentages. The comparison of normally distributed continuous variables between the groups was performed using Student’s t-test. Nominal categorical data between the groups were compared using the chi-squared test or Fisher’s exact test as appropriate. P < 0.05 was considered statistically significant.

Sample size calculation

Using the statistical software G*Power version 3.1.9.2, the sample size for this investigation was computed. In a study by Sale and Shendage [13], IV lignocaine was compared to IV dexmedetomidine for attenuation of hemodynamic response to laryngoscopy and intubation. The difference in the mean of MAP from baseline (91.00 ± 7.80) and one minute after intubation (80.50 ± 7.09) was used to calculate the effect size in this study. The effect size was 0.6, the power was 90%, and the alpha error was 0.05. The sample size was determined to be 98 patients, 49 in each group.

## Results

A total of 100 patients were randomised for the study, out of which 50 were nebulised with dexmedetomidine 1 microgram/kg made up to a volume of 5 ml by adding normal saline and the other 50 were nebulised with 5 ml saline.

Mean age, mean weight, mean duration of surgery, and time for intubation

It was observed that there was no significant difference in mean age (p = 0.920), mean weight (p = 0.377), mean duration of surgery (p = 0.645), and mean time for intubation (p = 0.709) between the two groups (Table 1).

**Table 1:** Comparison of mean age, mean weight, mean duration of surgery, and mean time for intubation between the two groups

	Treatment group (n = 50)			Control group (n = 50)			P-value
	Mean ± SD	Min - Max	Median (Q1-Q3)	Mean ± SD	Min - Max	Median (Q1-Q3)	
Age (years)	38.66 ± 13.907	14 - 65	37 (29 - 50.25)	39.28 ± 14.475	14 - 70	37 (28 - 49.75)	0.920
Weight (kg)	52.2 ± 11.258	29 - 78	50 (45 - 60)	53.58 ± 9.305	36 - 75	50 (47 - 60)	0.377
Duration of surgery (hours)	2.1 ± 2.3	1 - 11	1.5 (1.3 - 2)	1.68 ± 0.728	1 - 4	1.5 (1 - 2)	0.645
Time for intubation (seconds)	12.26 ± 2.311	10 - 15	12 (10 - 15)	12.42 ± 1.95	10 - 15	12 (10 - 15)	0.709

Gender distribution

It was observed that there was no significant difference in the sex distribution of the patients when compared between the two groups (p = 0.075) (Table 2).

**Table 2:** Comparison of gender distribution

Sex	Treatment group		Control group		P-value
	Frequency	%	Frequency	%	
Male	10	12%	18	36%	0.075
Female	40	88%	32	64%	
Total	50	100%	50	100%	

Comparison of systolic blood pressure

Table 3 shows that there is a statistically significant difference in the SBP of the two groups recorded before laryngoscopy (p = 0.019), after intubation (p = 0.007), after one minute of intubation (p < 0.001), after five minutes (p < 0.001), and after 10 minutes (p = 0.010) of intubation.

**Table 3:** Comparison of mean SBP at various time points between the two groups SBP: systolic blood pressure; SD: standard deviation; Min - Max: minimum - maximum.

SBP	Treatment group		Control group		P-value
	Mean ± SD	Min - Max	Mean ± SD	Min - Max	
Baseline	121.96 ± 13.046	97 - 152	122.39 ± 15.918	93 - 162	0.817
After nebulisation	118.98 ± 10.281	94 - 147	121.64 ± 15.48	92 - 160	0.314
Before laryngoscopy	117.58 ± 9.609	100 - 143	113.32 ± 14.038	86 - 145	0.019
After intubation	117.42 ± 12.119	100 - 155	126.29 ± 18.747	90 - 186	0.007
After 1 minute of intubation	113.2 ± 14.503	92 - 159	125.92 ± 15.263	97 - 160	<0.001
After 5 minutes of intubation	109.86 ± 8.342	92 - 125	118.2 ± 13.548	90 - 155	<0.001
After 10 minutes of intubation	114.24 ± 7.797	98 - 132	120.29 ± 14.12	95 - 152	0.010

Comparison of diastolic blood pressure

Table 4 shows the comparison of mean DBP at various time points between the two groups under the study. It was further observed that there was a significant difference in mean DBP at time intervals before laryngoscopy (p < 0.001), after intubation (p = 0.034), after one minute (p = 0.011), after five minutes (p < 0.005), and after 10 minutes (p = 0.009) of intubation when compared between the two groups; however, at baseline, no significant difference was observed between the two groups (p = 0.201).

**Table 4:** Comparison of mean DBP at various time points between the two groups DBP: diastolic blood pressure; SD: standard deviation; Min - Max: minimum - maximum.

DBP	Treatment group		Control group		P-value
	Mean ± SD	Min - Max	Mean ± SD	Min - Max	
Baseline	70.68 ± 8.702	51 - 93	73.32 ± 11.592	57 - 96	0.201
After nebulisation	76.76 ± 5.854	63 - 86	74.58 ± 11.53	56 - 98	0.236
Before laryngoscopy	76.76 ± 6.589	62 - 91	68.68 ± 12.011	51 - 102	<0.001
After intubation	75.2 ± 10.124	60 - 105	80.66 ± 14.752	54 - 104	0.034
After 1 minute of intubation	73.72 ± 10.986	55 - 101	79.18 ± 9.917	64 - 101	0.011
After 5 minutes of intubation	71.62 ± 9.934	48 - 86	77.48 ± 10.595	53 - 105	0.005
After 10 minutes of intubation	76.1 ± 8.006	63 - 93	81.38 ± 11.366	60 - 111	0.009

Comparison of mean blood pressure

Table 5 shows the comparison of MAP at various time points between the two groups under the study. It was further observed that there was a significant difference in the MAP at time intervals before laryngoscopy (p < 0.047), after intubation (p = 0.042), after one minute (p = 0.001), after five minutes (p < 0.006), and after 10 minutes (p = 0.018) of intubation when compared between the two groups; however, at baseline, no significant difference was observed between the two groups (p = 0.081).

**Table 5:** Comparison of mean MAP at various time points between the two groups MAP: mean arterial pressure; SD: standard deviation; Min - Max: minimum - maximum.

MAP	Treatment group		Control group		P-value
	Mean ± SD	Min - Max	Mean ± SD	Min - Max	
Baseline	87.74 ± 9.29	66 - 112	88.82 ± 14.28	60 - 110	0.081
After nebulisation	90.90 ± 6.48	73 - 100	90.26 ± 12.28	70 - 118	0.773
Before laryngoscopy	90.40 ± 7.01	75 - 107	85.88 ± 12.27	67 - 119	0.047
After intubation	89.28 ± 10.63	73 - 122	95.02 ± 16.56	48 - 130	0.042
After 1 minute of intubation	86.80 ± 11.86	67 - 120	94.82 ± 11.06	75 - 119	0.001
After 5 minutes of intubation	84.44 ± 8.97	63 - 98	90.18 ± 11.24	63 - 117	0.006
After 10 minutes of intubation	88.72 ± 7.44	75 - 106	93.52 ± 11.87	68 - 120	0.018

Comparison of heart rate

It was observed that the heart rate was attenuated in the treatment group in a statistically significant manner with the p-values before laryngoscopy (p = 0.015), after intubation (p = 0.024), after one minute (p = 0.001), after five minutes (p = 0.003), and after 10 minutes of intubation (p = 0.013) (Table 6).

**Table 6:** Comparison of mean heart rate at various time points between the two groups SD: standard deviation; Min - Max: minimum - maximum.

Heart rate	Treatment group		Control group		P-value
	Mean ± SD	Min - Max	Mean ± SD	Min - Max	
Baseline	85.84 ± 13.764	55 - 111	89.72 ± 17.963	55 - 130	0.228
After nebulisation	82.6 ± 11.759	59 - 105	88.74 ± 18.43	50 -128	0.050
Before laryngoscopy	81.56 ± 11.348	60 - 102	88.28 ± 15.422	60 - 123	0.015
After intubation	83.34 ± 12.146	66 - 104	89.22 ± 13.391	65 - 116	0.024
After 1 minute	83.34 ± 12.325	58 - 105	91.94 ± 12.255	66 - 114	0.001
After 5 minutes	81.56 ± 13.33	54 - 99	89.56 ± 12.591	65 - 118	0.003
After 10 minutes	80.16 ± 14.086	52 - 104	87.4 ± 14.445	59 - 138	0.013

Propofol used for induction

Table 7 shows the comparison of mean propofol used for induction between the two groups. Under the treatment group, it was observed that the mean propofol used for induction was 73 ± 19.509 mg, while in the control group, the mean was 105.2 ± 14.741 mg.

**Table 7:** Comparison of mean propofol used for induction between the two groups mg: milligram; SD: standard deviation; Min - Max: minimum - maximum; Q1 - Q3: lower quartile - upper quartile.

	Treatment group			Control group			P-value
	Mean ± SD	Min - Max	Median (Q1-Q3)	Mean ± SD	Min - Max	Median (Q1-Q3)	
Propofol used for induction (mg)	73 ± 19.509	50 - 120	70 (60 - 90)	105.2 ± 14.741	80 - 140	100 (100 - 120)	<0.001

It was further observed that there was a significant difference in the mean when compared between the two groups (p < 0.001). Nebulised dexmedetomidine given pre-operatively significantly reduced the dose of propofol used for induction during general anaesthesia.

Comparison of sedation

There was no sedation in both the groups according to the Observer's Assessment of Alertness/Sedation Scale. The level of sedation was noted in both groups after nebulisation was complete. All the patients responded readily to the name spoken in a normal tone, the speech was normal, facial expression was normal, and the eyes were clear with no ptosis. Hence, all patients in both the groups had a score of 5 on the Observer's Assessment of Alertness/Sedation Scale after nebulisation in both the groups.

## Discussion

In this prospective randomised study, we found significant attenuation of hemodynamic response to laryngoscopy and intubation in group A as compared to group B. There was no incidence of hypotension or bradycardia in either group A or B. This study was formulated to avoid the side effects of intravenous dexmedetomidine, i.e., hypotension and bradycardia, which are known adverse effects of intravenous dexmedetomidine.

Intubation and laryngoscopy can produce tachycardia, hypertension, laryngospasm, bronchospasm, increased intracranial pressure, and increased intraocular pressure [14]. Reid and Brace were the first to document the hemodynamic alterations that laryngoscopy and intubation cause [15]. The response begins within five seconds of laryngoscopy, peaks in one to two minutes, and recovers to normal in five minutes [14]. These alterations are normally transient, and healthy people bear them well. In patients with cardiovascular problems and cerebrovascular disease, these hemodynamic perturbations can lead to myocardial ischemia, ventricular dysrhythmias, ventricular failure, pulmonary oedema, as well as cerebrovascular accidents [14].

Dexmedetomidine is an alpha-2A receptor agonist. It acts on alpha-2A receptors located in locus coeruleus, the predominant noradrenergic nuclei of the upper brain stem, and it inhibits noradrenaline release. It has sedative, hypnotic, anxiolytic, sympatholytic, antisecretory, and analgesic properties. It has no respiratory depressant effect. Reduced sympathetic activity results from postsynaptic stimulation of alpha-2 receptors in the central nervous system, resulting in bradycardia and hypotension [16]. Atipamizole is the reversal drug for dexmedetomidine; it acts by increasing the central turnover of noradrenaline. Many studies have used intravenous dexmedetomidine to reduce the hemodynamic response to intubation; however, it has been found to cause hypotension and bradycardia [17]. Alternative routes for dexmedetomidine delivery are being investigated to avoid this. Intravenous dexmedetomidine in the doses of 0.5-1 microgram/kg has proved to be effective in various studies to decrease the intensity of hemodynamic response to intubation [18-20]. So this study was designed to study whether dexmedetomidine when given via nebulisation route can attenuate hemodynamic response to laryngoscopy and intubation or not. When given via the nebulisation route, the nasal mucosa accounts for 65% of dexmedetomidine bioavailability, whereas the buccal mucosa accounts for 82% [10].

Only two randomised controlled trials have been done to study the effect of nebulised dexmedetomidine on attenuation of hemodynamic response to laryngoscopy and intubation. The study done by Kumar et al. [8] concluded that nebulised dexmedetomidine decreases the hemodynamic response to laryngoscopy and intubation with no adverse effects. A low dose of propofol was required during induction in the treatment group.

Another study done by Misra et al. [9] concluded that nebulised dexmedetomidine decreased the increase in heart rate but not SBP during and after laryngoscopy and intubation. There was a considerable reduction in the dose of propofol required at induction of general anaesthesia in the treatment group. Misra et al. [9] found that intraoperative requirement of fentanyl and isoflurane also decreased significantly in the nebulised dexmedetomidine group.

There was significant attenuation of heart rate, SBP, DBP, and mean blood pressure at one minute, five minutes, and 10 minutes following intubation in the group of patients who received dexmedetomidine nebulisation in the present study. Nebulised dexmedetomidine has a dose sparing effect on the induction dose of propofol.

## Conclusions

Nebulisation with dexmedetomidine 1 microgram/kg attenuates the hemodynamic response to laryngoscopy and intubation, without the incidence of hypotension and bradycardia. It is a new route of administration for reducing the hemodynamic response to laryngoscopy and intubation, so further research is required in this field. No sedation was observed in either group A or group B patients, according to the Observer's Assessment of Alertness/Sedation Scale. Nebulised dexmedetomidine appears to be a promising drug to attenuate the hemodynamic response to laryngoscopy and intubation.
